# Dataset of companies’ profitability, government debt, financial statements' key indicators and earnings in an emerging market: Developing a panel and time series database of value-added tax rate increase impacts

**DOI:** 10.12688/f1000research.132949.1

**Published:** 2023-04-14

**Authors:** Mahfoudh Mgammal, Ebrahim Al-Matari

**Affiliations:** 1 Accounting Department, College of Business, Jouf University, Sakaka, Saudi Arabia; 2 Amran University, Amran, Yemen

**Keywords:** Earnings; Government Debt; Performance; Taxation; Shareholders' Equity; Profitability

## Abstract

Company profitability is a crucial indicator that can be used for developing and sustaining trust in accounting information and, thus, inefficient capital markets. Companies with good financial statements’ key indicators have a more extensive customer base and can diversify their revenue streams, making them more resilient to economic downturns. Assembling and managing taxes is a critical underpinning to protecting a country’s financial intensity and developing a country’s tax-system. VAT is a primary source of financial gain in developing nations, which differs from economic income in developed countries, where economic income is primarily derived from tax income. In emerging economies, the existing practice requires firms to effectively and efficiently publish annual-reports and indicators on market-websites, as users rely heavily on timely-information and need it to make decisions. However, these practices fell short of expectations, requiring more research. These variables are crucial for most accounting/economics/taxation research models and the lack of easily attainable data in well-known databases (e.g., ARGAAM; DataStream). This article is primarily a dataset for analysing taxation, performance variables, and key financial-statement indicators. The data describes the raw, combined, and filtered information at the company level, such as company profit and government debt in Saudi Arabia. It combines a firm-level panel dataset sample of company profit that its measures scaled by total assets and include: earnings before interest, taxes, decrease and amortisation, earnings before interest and taxes, earnings after taxes and earnings before taxes—moreover, the time series dataset sample of 11 financial statements’ key indicators. The dataset results from 494 company-year observations (226-panel data sample and 268-time series data sample) from 2019 to 2020. Data has been collected from taxation reports, corporate annual reports, ARGAAM database, FinBox database, the Trading Economics database and the Tadawul-market website in Saudi Arabia.

## Introduction

The reasoning and setting behind the creation of this dataset are to help the researcher to perform a comparative analysis across different disciplines to study the role of these variables in a specific phenomenon or the factors enlightening value-added tax (VAT) usefulness and effectiveness. For example, estimate the consequences of a VAT rate increase on profitability, government debt etc. This dataset helps complete two of the few studies investigating the VAT effect in the Kingdom of Saudi Arabia (KSA). There was an encouragement to observe the effect of imposing the new 15% VAT on the profitability of nonfinancial Saudi-listed companies. This dataset is in two types: a firm-level panel dataset sample and a time series sample ( Mgammal, 2021; Mgammal, Al-Matari, and Alruwaili, 2023).

## Methods

This dataset article contains proxies to measure the economic impacts of VAT as used by several prior researchers ( Mgammal, 2021). The representatives for measuring the economic effects of VAT were manually collected and built using secondary data obtained from publicly available data from 2019 to 2020. In Table 1, ProFtEBITDA means company profit measured by earnings before interest, taxes, devaluation, and amortisation (EBITDA) and scaled by total assets and data collected from FinBox database tools. Data were hand collected from companies’ tax reports. We specify companies and identify them with inclusion/exclusion criteria, as mentioned in Table 3. The inclusion/exclusion criteria of all data in this article are as follows: we included nonfinancial companies and excluded finance firms. Then we filtered the sample by excluding companies with annual reports unavailable for two years. The fiscal year-end date is not 31/12/of each year, and the accounting period is over 12 months. Consequently, the final dataset is 494 company-year observations (226-panel data sample and 268-time series data sample) from 2019 to 2020. This final sample is the foundation that can be used for analysis in future research. SIZE2020 and SIZE2019 are the mean company size in 2020 and 2019, measured by the natural logarithm of total assets and data gathered from companies’ annual reports. EAT2020 and EAT2019 are the mean company earnings after tax in 2020 and 2019 and were measured by deducting all expenditures and revenue taxes from the business’s revenues. Following prior research, two variables were added, GvD2020 and GvD2019, which meant government debt in 2020 and 2019 and was measured as government debt over the gross domestic product (GDP). Data for this were extracted from the Trading Economics ( https://tradingeconomics.com/) database. Regarding the process for accessing the data, the data was hand collated directly from the Trading Economics website using many Saudi Arabia Indicators reports, especially GDP Indicators reports. In this context, the data collected from the Trading dataset are the same inclusion/exclusion criteria as mentioned above, and we started data collection on 23/01/2021 to 30/03/2021.

**Table 1.** Definitions of the variables.

Variables	Definition
ProFtEBITDA	Company Profit
SIZE2020	Company Size in 2020
EAT2020	Company Earnings after tax in 2020
GvD2020	Government Debt 2020
EAT2019	Company Earnings after tax in 2019
SIZE2019	Company Size in 2019
GvD2019	Government Debt 2019

As in Table 2 below, an index containing 11 items was included: BALANCE SHEET: total assets, total equity, and liabilities-equity. INCOME STATEMENT: total income, total revenues, total expenses, and net income. CASH FLOW: changes in operation. Activity, changes in investing act, changes in the financing act, and cash at the end of the period. These factors help control for potential impacts when analysing how a VAT increase will affect a firm. We take each into account as each has a component of the likely effect of a VAT increase. Collectively, these classifications were based on data available on the Tadawul market website as we clarify in inclusion/exclusion criteria above. It is a periodic data set of most Saudi registered companies from before the introduction of the new VAT rate of 15% in 2019 to after the introduction of the new VAT rate in 2020. The dataset framework was chosen when the new 15% VAT was introduced due to public access to VAT information for nonfinancial companies. Tadawul requires all public companies to publish their financial statements on the Tadawul website quarterly and annually ( https://m5.gs/OXFqam) (Tadawul, 2019-2020). These data in the file can help build additional variables, such as measuring the effect of VAT before and after increasing its rate using unique techniques such as the difference-in-difference (DID) approach and the autoregressive integrated moving average (ARIMA) modelling approach. Nevertheless, since the literature does not provide common definitions or metrics, we leave the creation of these additional variables at the discretion of potential users. Thus, users can use this dataset to create these measurements from their perspective ( Baatwah and Aljaaidi, 2021; Mgammal, 2021).

**Table 2.** Financial statements’ key indicators definitions.

No	Variables abbreviations	Before (b) in 2019	After (a) in 2020
		**Balance sheet**	**Balance sheet**
**1**	TAb & TAa	1. Total Assets (TAb)	1. Total Assets (TAa)
**2**	SEb & SEa	2. Shareholders’ Equity (SEb)	2. Shareholders’ Equity (SEa)
**3**	TLSEb & TLSEa	3. Total Liabilities and Shareholder Equity (TLSEb)	3. Total Liabilities and Shareholder Equity (TLSEa)
		**Statement of income**	**Statement of income**
**4**	TIb & TIa	4. Total Income (TIb)	4. Total Income (TIa)
**5**	TRb & TRa	5. Total Revenues (TRb)	5. Total Revenues (TRa)
**6**	TEb & TEa	6. Total Expenses (TEb)	6. Total Expenses (TEa)
**7**	NIb & NIa	7. Net Income (NIb)	7. Net Income (NIa)
		**Cash flow**	**Cash flow**
**8**	COAb & COAa	8. Other Changes in Oper. Activity (COAb)	8. Other Changes in Oper. Activity (COAa)
**9**	CIAb & CIAa	9. Other Changes in Investing Act. (CIAb)	9. Other Changes in Investing Act. (CIAa)
**10**	CFAb & CFAa	10. Other Changes in Financing Act. (CFAb)	10. Other Changes in Financing Act. (CFAa)
**11**	CEPb & CEPa	11. Cash at End of Period (CEPb)	11. Cash at End of Period (CEPa)

### Data description

The dataset included in this article contains three files describing and defining the sample and variables. Excel file 1 consists of all raw and filtered data for the variables for the panel data sample. Excel file 2 depicts time-series and cross-sectional data for nonfinancial firms listed on the Saudi market for the second and third quarters of 2019 and the third and fourth quarters of 2020. Excel file 3 presents the raw material of variables used in measuring the company’s profitability of the panel data sample. The period of this data is selected from an extensive section of registered companies in Saudi Arabia from 2019 before imposing the new VAT rate, 15%, to 2020 after setting the new VAT rate. The major segments are consumer discretionary, information technology, energy, consumer staples, materials, health care, industrials, communication services, real estate, utilities, and financials. The sample framework was chosen due to the time of implementation new 15% VAT rate and the public access to information about VAT if nonfinancial companies, where Tadawul forces all listed companies to publish their financial statements publicly quarterly and annually on the Tadawul website. Financial companies were excluded from the sample framework as they have unique treatments, and some previous studies investigated the effects of VAT on KSA Banks. The final panel data sample framework is 131 listed companies and 268 observations for the time sires’ sample, as depicted in Table 3 below.

**Table 3.** Descriptive for the samples’ characteristics.

Sectors	VAT 5% implementation period *	VAT 15% Implementation period *	Panel data sample by companies	Time-series sample by quarterly observations
Energy	From Jan. 1, 2018, to July 1, 2020	From July 1, 2020, ongoing	5	10
Materials			42	84
Commercial and Professional Services			3	6
Transportation			5	10
Consumer durables and Apparel			6	12
Consumer services			10	20
Media and entertainment			2	4
Retailing			8	16
Food and staples retailing			4	4
Food and beverages			12	24
Health care equipment and Service			7	14
Pharma, Biotech and Life Science			1	2
Software and services			2	4
Telecommunication services			4	8
Utilities			2	4
Capital goods			-	24
Real estate management and development			-	22
Total			113*2 = 226 Obs	268 Obs

* In the Kingdom of Saudi Arabia (KSA), value-added tax (VAT) was first introduced in all industries as a 5% VAT on goods and services as of Jan. 1, 2018, and, because of COVID-19, the Kingdom of Saudi Arabia (KSA) increased the VAT from 5% to 15% on July 1, 2020.

We utilise balanced panel data as it is a more sensitive measurement of the modifications that could occur between points in time ( Cavana, Delahaye, & Sekeran, 2001). Additionally, the outcomes created are more robust, consistent, and stable, enabling a generalisation of the population to be more meaningful and representative. Therefore, the final dataset sample is 226 observations were specified to be eligible for implication in the analyses. It is the basis for the research, i.e. multivariate, bivariate, additional tests and descriptive.

### Descriptive statistics

To recognise and determine the situation of every concept, descriptive statistics were utilised to clarify. Table 4 displays the statistics of descriptive (standard deviation, median, mean, minimum, maximum values and degrees of freedom) for 113*2 = 226 observations of all variables of the panel data sample and 268 observations for the time series sample.

**Table 4.** Descriptive statistics.

(A) Penal data sample (Observations = 226)					
Variable	Obs	Mean	Std. Dev.	Min	Max
ProFtEBITDA	113	0.0000475	0.0000871	-0.0002105	0.000412
ProFtEBIT	113	0.0000227	.0000751	-0.000222	0.0001892
ProFtEBTUnu	113	0.0000344	.0001461	-0.0002215	0.0013329
**2019 (Before Tax Reform)**					
EAT2019	113	0.0000842	0.0007884	-0.0006502	0.008303
SIZE2019	113	6.402416	0.7762833	4.799299	9.600352
GvD2019	113	2.224271	8.346027	0.0095256	7.217121
**2020 (After Tax Reform)**					
EAT2020	113	0.0000171	0.0000853	-0.0002951	0.0002637
SIZE2020	113	6.401014	0.7646444	4.63827	9.274225
GvD2020	113	1.27e+14	7.60e+14	1.79e+11	7.75e+15
Chg (ERN)	113	2.973124	25.42851	-16.4361	264.1

(B) Time series data sample (Observations = 268)									
Before (b) in 2019					After (a) in 2020				
Variable	Mean	Std. Dev.	Min	Max	Variable	Mean	Std. Dev.	Min	Max
**OTAb**	1.28E+07	5.08E+07	62994	4.77E+08	**TAa**	1.28E+07	5.17E+07	43478	5.03E+08
**SEb**	4788402	1.69E+07	55457	1.69E+08	**SEa**	4652292	1.66E+07	14494	1.65E+08
**TLSEb**	1.28E+07	5.08E+07	62994	4.77E+08	**TLSEa**	1.28E+07	5.17E+07	43478	5.03E+08
**TIb**	331645.6	1109441	-3407	8375361	**TIa**	318024.6	1206166	-242725	8970955
**TRb**	347839.8	1140208	-105027	8699826	**TRa**	338827.1	1254699	-178025	9083262
**TEb**	233783.7	980947.3	-1374325	7746953	**β17TEa**	225897.5	996286.7	-1199752	7762214
**NIb**	78985.93	356338.2	-724512	2679387	**NIa**	62371.49	342734.2	-609803	2765537
**COAb**	55342.46	970096.6	-4252574	9451664	**COAa**	86007.48	600282.1	-1329988	6184696
**CIAb**	191045.9	1078954	-455858	7620294	**CIAa**	15994.71	268631.9	-1140654	1589257
**CFAb**	-642943	2665571	-2.03E+07	517795	**CFAa**	-439313	1907536	-1.57E+	278246
**CEPb**	698759	3357006	-4764	3.74E+07	**CEPa**	619081.7	2733571	-34220	3.02E+07

### Value of the data

• This dataset is essential because it covers data on variables rarely overlooked in accounting, taxation and business performance research models, collectively or individually, but appeal to a wide range of stakeholders. For example, it enables capital markets regulators, standard setters, practitioners and users of financial reporting to easily access long-term data to assess the effectiveness and efficiency of VAT in controlling the risk of tax evasion in fast-growth markets.
• This dataset is valuable for interdisciplinary studies investigating the role of these variables in specific phenomena or factors enlightening VAT and company performance effectiveness.
• This dataset is beneficial because it contains data on profitability collected to measure company profit using earnings before interest, taxes, depreciation, and amortisation (EBITDA) and scaled by the total assets data set. Further, the dataset has been arranged into individual and multiple measurements.
• The data allows researchers to scrutinise the influence of VAT increase on various accounting matters, such as corporate governance mechanisms, the performance of companies and the quality of financial reporting. Furthermore, this dataset is valuable to related parties, e.g., investors, stakeholders, tax authorities, decision-makers, managers and market regulators in assessing and reviewing the tax system in Saudi Arabia. This assessment will give them assurance in making different decisions.
• These data can be analysed and/or compared to other emerging economies and G20 countries ( https://www.g20.org/en/). It can be used in discussing the tax system and VAT rate regarding which parties are responsible for adding some VAT incentives in the tax system and updating the tax system sideways with VAT implementation to advantage from the effectiveness of VAT in the KSA. Because KSA could be similar to other countries in G20 and Gulf Cooperation Council (GCC).
• The data is also beneficial for studies on VAT incentives efficiency using data in the long term.

## Data Availability

Harvard Dataverse: Dataset of companies’ profitability, government debt, Financial Statements’ Key Indicators and earnings in an emerging market: Developing a panel and time series database of value-added tax rate increase impacts. https://doi.org/10.7910/DVN/HEL3YG ( Mgammal and Al-Matari, 2023). The project contains the following underlying data:
• Final Data - Panel dataset- DiB-2.xlsx. (Excel file 1 consists of all raw and filtered data for the variables for the panel data sample). • VAT-Final-Time seires dataset DIB-2.xlsx (Excel file 2 depicts time-series and cross-sectional data for nonfinancial firms listed on the Saudi market for the second and third quarters of 2019 and the third and fourth quarters of 2020). • Raw matiral – DiB-2.xlsx. (Excel file 3 presents the raw material of variables used in measuring the company’s profitability of the panel data sample). Data are available under the terms of the Creative Commons Attribution 4.0 International license (CC-BY 4.0).
