# Supplementary material for: Dataset of companies’ profitability, government debt, financial statements' key indicators and earnings in an emerging market: Developing a panel and time series database of value-added tax rate increase impacts
Source: F1000Res. 2023 Apr 14;12:405. [Version 1] doi: 10.12688/f1000research.132949.1 (PMC10905151; doi:10.12688/f1000research.132949.1)
Supplement: Peer review history

# Reviewer report: Reviewer response for version 1

**DOI:** 10.5256/f1000research.145908.r230650

**Published:** 2024-02-08

**Authors:** Faozi Almaqtari

**Affiliations:** 1 A'Sharqiyah University (ASU), Ibra, Oman

Thank you for giving me chance review of this intriguing data article. I believe that by utilizing this dataset, researchers can explore compelling issues within the realm of value-added tax (VAT) in the specific context of Saudi Arabia. I would kindly suggest that the authors carefully review the paper, as there are a few minor typos that could be addressed. In summary, I anticipate significant value emanating from this dataset, foreseeing its potential to contribute valuable insights and implications for economic considerations.

Are sufficient details of methods and materials provided to allow replication by others?

Yes

Is the rationale for creating the dataset(s) clearly described?

Yes

Are the datasets clearly presented in a useable and accessible format?

Yes

Are the protocols appropriate and is the work technically sound?

Yes

Reviewer Expertise:

Governance, Financial Performance, Auditing

I confirm that I have read this submission and believe that I have an appropriate level of expertise to confirm that it is of an acceptable scientific standard.

# Reviewer report: Reviewer response for version 1

**DOI:** 10.5256/f1000research.145908.r172936

**Published:** 2023-05-24

**Authors:** Saeed Rabea Baatwah

**Affiliations:** 1 Department of Accounting, College of Business Administration, Shaqra University, Shaqra, Riyadh Province, Saudi Arabia

Thank you for reviewing this interesting data article. I think that researchers can test interesting issues in the context of VAT and in Saudi Arabia using this dataset. I would suggest to the authors that it will be more reflective of the dataset if the title is updated to "Dataset of value-added tax, companies’ profitability, government debt, financial statements' key indicators, and earnings in an emerging market: Developing a panel and time series dataset". Furthermore, I would suggest the authors review the paper, as there are some minor typos. Overall. I expect greater value from this dataset and its potential to help draw economic implications.

Are sufficient details of methods and materials provided to allow replication by others?

Yes

Is the rationale for creating the dataset(s) clearly described?

Yes

Are the datasets clearly presented in a useable and accessible format?

Yes

Are the protocols appropriate and is the work technically sound?

Yes

Reviewer Expertise:

I am interested in auditing, financial reporting, CSR, taxation, and emerging countries.

I confirm that I have read this submission and believe that I have an appropriate level of expertise to confirm that it is of an acceptable scientific standard.

# Response

**Published:** 2023-05-31

**Authors:** Mahfoudh Mgammal

**Affiliations:** Accounting, Amran university, Amran, Amran, Yemen

I would like to thank you for this great feedback and suggestions. We agree with you that updating the title to "Dataset of value-added tax, companies’ profitability, government debt, financial statements' key indicators, and earnings in an emerging market: Developing a panel and time series dataset" would be more reflective of the dataset. It's also good to hear that the dataset has the potential to draw economic implications. Moreover, we confirmed that we reviewed and corrected the paper for minor typos as per requested. In this regard, we looking forward to seeing the greater value that this dataset can bring to researchers studying VAT and Saudi Arabia.
